# Medical student attitudes and perceptions of psychedelic-assisted therapies

**DOI:** 10.3389/fpsyt.2023.1190507

**Published:** 2023-06-27

**Authors:** Irene Li, Rodney Fong, Molly Hagen, Burton Tabaac

**Affiliations:** ^1^School of Medicine, University of Nevada, Reno, Reno, NV, United States; ^2^School of Public Health, University of Nevada, Reno, Reno, NV, United States; ^3^Department of Neurology, Carson Tahoe Health, Reno, NV, United States

**Keywords:** psychedelics, perceptions, attitudes, opinions, medical students, psilocybin, LSD, DMT

## Abstract

**Introduction:**

Although certain psychedelic agents may soon gain federal approval for use in treating specific psychiatric conditions, the utilization of such therapies in clinical practice will depend largely on the attitudes of healthcare providers. Therefore, this study assesses the current attitudes, knowledge, exposure, and acceptance of psychedelics and psychedelic-assisted therapies amongst medical students.

**Methods:**

In fall semester of 2022, surveys were emailed to 580 medical students attending medical institutions in the state of Nevada in the United States. Utilizing knowledge and attitude items from previously published studies, the survey collected demographic data and assessed student attitudes with five-point Likert-scale variables. Data was analyzed using summary statistics and Kruskal-Wallis tests for differences in mean survey scores (i.e., attitudes towards psychedelics) based on demographic factors.

**Results:**

132 medical students participated in the survey (22.7% response rate). Medical students demonstrated overall positive attitudes towards psychedelics, lack of knowledge regarding psychedelics, and uncertainty towards neurocognitive risks of psychedelics. Overall, 78.6% of students agreed that psychedelics have therapeutic potential, while 95.2% agreed that psychedelics deserves further research in assessing this potential. Additionally, there was no statistically significant effect of demographic variables, including age, sex, and level of training, on attitudes.

**Discussion:**

Although students are overall curious and optimistic about psychedelics, they demonstrate a lack of knowledge regarding recent research efforts. As the field of psychiatry prepares to implement psychedelics and psychedelic-assisted therapies, education and awareness of such agents should be initiated early on in medical clinical training.

## 1. Introduction

Psychedelic drugs, such as psilocybin and lysergic acid diethylamide, have been historically stigmatized in the United States. Recent years, however, have served as a renaissance of renewed interest and dedicated research regarding the use of psychedelics as potential agents in the treatment of psychiatric maladies (1–3). The early data suggests utility in hallucinogenic agents for treating anxiety, depression, nicotine use disorder, alcohol use disorder, and post-traumatic stress disorder (PTSD), amongst other conditions (4–9). Given the growing compelling evidence, the field of psychiatry has been suggested to start preparing for the possibility of Food and Drug Administration (FDA) approval in the near future of psychedelics and psychedelic-assisted therapies, most notably 3,4-methylenedioxymethamphetamine (MDMA) for the treatment of PTSD (10, 11).

In light of these recent developments, the current attitudes and perceptions of psychedelics and psychedelic-assisted therapies have been assessed in a myriad of demographic populations, including psychiatrists, psychologists, counselors, cancer healthcare workers, palliative care providers, college students, mental health service patients, individuals with eating disorders, and individuals with fibromyalgia (11–25). As of 2016, psychiatrists perceived psychedelics as potentially hazardous and appropriately illegal for recreational purposes, with less than half of those surveyed (42.5%) believing that psychedelics showed promise in treating psychiatric disorders (11, 12). These attitudes may vary based on nationality, culture, and time, as psychiatrists in the United Kingdom in 2021 demonstrated a larger proportion in agreement (77.2%) towards the therapeutic role of psychedelics (21). However, there appears to be broad support for further research into psychedelics amongst both healthcare workers and patients alike. For example, palliative care providers believe psychedelic-assisted therapies may aid in treating refractory existential distress (19), while individuals with fibromyalgia reported willingness to participate in a clinical trial if one existed. Overall, the majority of demographic groups mentioned in their respective study supported additional research into psychedelic-assisted therapies.

To date, there is scarce literature on the beliefs among medical students regarding psychedelic agents, thus limiting the understanding of awareness among a critical cohort of the healthcare workforce. The only study thus far on medical student attitudes focused on psilocybin specifically, finding significantly positive perceptions amongst medical students with greater perceived knowledge of medical psilocybin, less concern for possible adverse effects, and greater belief in the recreational legalization of psilocybin (26). However, further exploration is required to better understand the attitudes of medical students towards all forms of psychedelic-assisted therapies. Given that medical students are in the nascent stages of their clinical training, it is probable that this group of trainees will witness the implementation and dissemination of drugs currently in a restricted group of therapeutics to transition into clinical practice, pending FDA approval. As scientific and cultural conversations evolve, it is essential to obtain a baseline assessment of medical students’ attitudes of all psychedelic agents in general, as their perceptions may influence clinical implementation and application of psychedelics in coming years. Whether psychedelics or psychedelic-assisted therapies become broadly utilized within psychiatric care and general medicine will depend not only upon the results of larger clinical trials, but also the attitudes of clinicians in a position to recommend such therapeutics. Understanding current trends of acceptance for psychedelic use among physicians-in-training has implications for both the future training and education of medicine.

This study seeks to investigate the awareness, knowledge, and attitudes of medical students in the United States regarding psychedelic-assisted psychiatric treatment. The authors posited that the majority of medical students would report optimism about the therapeutic potential of psychedelics and psychedelic-assisted therapies due to a younger demographic of student population and likely greater exposure to recent popular media publications about psychedelics in a favorable light. In line with the attitudes found amongst American psychiatrists (12), we hypothesized that younger age, male sex, and increased exposure to media publications is associated with opinions more favorable to the therapeutic potential of psychedelics and opinions more skeptical of adverse outcomes of psychedelics. As medical students are actively engaged in the process of electing a career specialty, we hypothesized that specific specialty preferences, such as psychiatry and neurology, may correlate with more favorable attitudes.

## 2. Methods

### 2.1. Survey design

The study survey consisted of an anonymous online 19-item Qualtrics® questionnaire. This consisted of three demographic variables (age range, gender, race/ethnicity) and posed inquiries of participants regarding which medical school they are attending, the type of medical school training they are receiving (allopathic versus osteopathic), their level of medical school training (MS1, MS2, MS3, MS4), and their current anticipated specialty of medicine (as a fill-in response).

Seven items were implemented to assess general attitudes and beliefs of psychedelics amongst enrolled participants, and each item was measured using a 5-point Likert scale ranging from “strongly disagree” to “strongly agree” (Supplementary Appendix A) based on a previous published study investigating attitudes of psychiatrists towards psychedelics (12). One item from a study focused on attitudes of college students (22) was incorporated to assess baseline knowledge of participants regarding psychedelic agents (e.g., “I would say I am knowledgeable about psychedelics”). One item was applied to assess sources participants have integrated to inform themselves about psychedelics (e.g., news media, social media, podcasts). Due to varying definitions of psychedelics, the questionnaire defined “psychedelic substances” as 5-MeO-DMT (5-methoxy-N,N-dimethyltryptamine), ayahuasca, DMT (N,N-Dimethyltryptamine), LSD (Lysergic acid diethylamide), psilocybin, and MDMA (3,4-methylenedioxy-methamphetamine), abstracted from the definition highlighted in a study investigating psychiatrists’ attitudes (12, 14).

### 2.2. Study sample

Medical students were surveyed in fall semester of 2022 from three schools located in the state of Nevada in the United States: University of Nevada, Reno School of Medicine; Kirk Kerkorian School of Medicine at University of Nevada, Las Vegas; and Touro University Nevada College of Osteopathic Medicine. The former two schools are allopathic, and the latter school is osteopathic in training. Among the allopathic institutions, students were recruited through communications sent to university affiliated email addresses, distributed to all levels of class listservs (first-year medical student, MS1; second-year medical student, MS2; third-year medical student, MS3; fourth-year medical student, MS4). Data collated from the osteopathic institution recruited students *via* specific research survey distribution listserv; per school policy, students could only be reached if they had previously indicated an interest in receiving research surveys. Communication emails were sent using secure, university-provided invitations, and restricted to verified email accounts. The recruitment emails included information about the study and a secure link to a Qualtrics® survey. Inclusion criteria included: (1) Age 18 years or older, (2) Current MS1, MS2, MS3, or MS4, and (3) Currently enrolled in active courses. The study was deemed exempt and approved by the University of Nevada, Reno School of Medicine Institutional Review Board.

### 2.3. Data analysis

All summary statistics and inferential tests were carried out in the program R (R Foundation for Statistical Computing, Vienna, Austria). Survey entries were excluded from summary statistics and inferential tests for the reasons of survey non-response, in which surveys were launched by no data was entered, and item non-response, in which only demographic data was provided but attitude questions were left blank. In all cases significance was assessed using *p* = 0.05.

Likert-scale variables were converted to numerical equivalents. Coding was assigned to Likert-scale variables to ensure that higher numbers indicated more positive views towards psychedelics in medicine. Specifically, survey question numbers # and #-# were coded as “Strongly disagree = 1” to “Strongly agree = 5,” and survey question numbers #-# were coded as “Strongly disagree = 5” to “Strongly agree = 1” (Supplementary Appendix A). Numerical equivalents were used to tabulate summary statistics for each question individually, and the eight Likert-scale questions were summed to create an overall survey score which was tested against demographic factors using inferential tests. The range of possible mean scores was 8–40, with 8 indicating strongly negative attitudes, 40 indicating strongly positive attitudes, and a score of 24 as overall neutral. Because the number of individuals within each level of the demographic factors varied and because the data showed non-normal trends, we elected to use a Kruskal-Wallis test rather than a one-way ANOVA to evaluate differences in mean survey scores (i.e., attitudes towards psychedelics).

## 3. Results

### 3.1. Demographics

The questionnaire was distributed to 580 medical students, of which 132 individuals responded to the full survey and were included in this study (22.7%; Table 1). For the osteopathic institution that required students to elect interest in research survey participation prior to receiving survey emails, only 5 of those students (8.3%) responded. In total, 42.2% (*n* = 56; Table 1) of the sample identified as male, 56.1% (*n* = 74; Table 1) as female, and 1.5% (*n* = 2; Table 1) as non-binary. The majority of respondents were White (*n* = 93; 70.5%; Table 1), while 13.6% (*n* = 18; Table 1) self-reported as Asian, 1.5% (*n* = 2; Table 1) as Black, and 6.8% as multi-racial (*n* = 9; Table 1). Just over half of survey participants were aged between 25 and 34 years (*n* = 80; 60.6%; Table 1) while 34.9% were aged 24 years old or younger (n = 46; Table 1), and 4.6% (*n* = 6; Table 1) were aged 35 or older. Most respondents attended an allopathic medical school (95.5%, *n* = 126; Table 1), while only 3.8% (*n* = 5; Table 1) attended an osteopathic medical school. Respondents represented all four curriculum years, with 28.0% first-years (*n* = 37; Table 1), 26.5% second-years (*n* = 35; Table 1), 35.6% third-years (*n* = 47; Table 1), and 9.1% fourth-years (*n* = 12; Table 1). See Table 1 for demographic details.

**Table 1:** Demographics of overall survey participants.

Characteristic	N	%
**Age**		
18–24 years old	46	34.9%
25–34 years old	80	60.6%
35–44 years old	5	3.8%
45–54 years old	1	0.8%
**Gender**		
Male	56	42.2%
Female	74	56.1%
Non-binary/third gender	2	1.5%
**Race**		
White	93	70.5%
Black	2	1.5%
Asian	18	13.6%
Multi-racial	9	6.8%
Prefer not to answer	10	7.6%
**Ethnicity**		
Hispanic/Spanish/Latino	25	18.9%
Non-Hispanic	104	78.8%
Prefer not to answer	3	2.3%
**Medical School**		
UNR^a^	104	78.8%
UNLV^b^	22	16.7%
TUNVOM^c^	5	3.8%
Prefer not to answer	1	0.8%
**Medical school type**		
M.D./ Allopathic	126	95.5%
D.O./ Osteopathic	5	3.8%
Prefer not to answer	1	0.8%
**Year in program**		
First	37	28.0%
Second	35	26.5%
Third	47	35.6%
Fourth	12	9.1%
Prefer not to answer	1	0.8%

*N* = sample size, % = percentage of sample for each level of the characteristic.

a University of Nevada, Reno School of Medicine.

b Kirk Kerkorian School of Medicine at UNLV.

c Touro University Nevada College of Osteopathic Medicine.

### 3.2. Attitudes

We calculated summary statistics for student attitudes using M.D. students only due to the small number of D.O. students (n = 5) that participated. We also excluded one student who did not identify the program type (M.D./D.O.) in which he/she was enrolled. Responses to Likert-scale opinion questions are shown in Table 2. Of the 126 M.D. survey participants, 40.5% agreed or strongly agreed that they were “knowledgeable about psychedelics.” Perceived negative effects of psychedelic use were relatively neutral regarding risk for subsequent psychiatric disorders (mean = 3.0; SE = 0.09; Table 2) and risk of long-term cognitive impairment (mean = 3.23; SE = 0.09; Table 2). In regards to the legality of psychedelics, only 21.4% (*n* = 27) agreed or strongly agreed with the illegality of psychedelic use for recreational purposes. Furthermore, 80.2% disagreed or strongly disagreed that psychedelic use remains unsafe even under medical supervision. In terms of therapeutic potential, 83.3% agreed or strongly agreed that the “the use of psychedelics may improve outcomes if used adjunctively with psychotherapy.” Additionally, 95.2% agreed or strongly agreed that psychedelics “deserves further research” to assess potential for treating psychiatric disorders.

**Table 2:** Overall summary statistics for attitudes towards psychedelic substances among MD students.

Survey question	Strongly disagree (1)	Somewhat disagree (2)	Neither (3)	Somewhat agree (4)	Strongly agree (5)	Mean	SE	IQR
I would say I am knowledgeable about psychedelics.	*n* = 30, 23.8%	*n* = 28, 22.2%	*n* = 17, 13.5%	*n* = 43, 34.1%	*n* = 8, 6.3%	2.77	0.12	2–4
The use of psychedelics increases the risk for subsequent psychiatric disorders.	*n* = 3, 2.38%	*n* = 40, 31.75%	*n* = 39, 31.0%	*n* = 37, 29.4%	*n* = 7, 5.6%	3.04	0.09	2–4
The use of psychedelics increases the risk of long-term cognitive impairment.	*n* = 15, 11.9%	*n* = 35, 27.8%	*n* = 41, 32.5%	*n* = 34, 27.0%	*n* = 1, 0.8%	3.23	0.09	2–4
The use of psychedelics should be illegal for recreational purposes.	*n* = 31, 24.6%	*n* = 40, 31.7%	*n* = 28, 22.2%	*n* = 16, 12.7%	*n* = 11, 8.7%	3.51	0.11	3–4
The use of psychedelics is unsafe even under medical supervision.	*n* = 55, 43.7%	*n* = 46, 36.5%	*n* = 19, 15.08%	*n* = 5, 4.0%	*n* = 1, 0.8%	4.18	0.08	4–5
The use of psychedelics shows promise in the treatment of psychiatric disorders.	*n* = 3, 2.4%	*n* = 2, 1.6%	*n* = 22, 17.5%	*n* = 47, 37.3%	*n* = 52, 41.3%	4.14	0.08	4–5
The use of psychedelics may improve outcomes if used adjunctively with psychotherapy.	*n* = 1, 0.8%	*n* = 2, 1.6%	*n* = 18, 14.3%	*n* = 54, 42.9%	*n* = 51, 40.5%	4.21	0.07	4–5
The use of psychedelics deserves further research as potential treatment for psychiatric disorders.	*n* = 0, 0.0%	*n* = 2, 1.6%	*n* = 4, 3.2%	*n* = 31, 24.6%	*n* = 89, 70.63%	4.64	0.06	4–5

“Substances” defined as 5-MeO-DMT (5-methoxy-N,N-dimethyltryptamine), ayahuasca, DMT (N,N-Dimethyltryptamine), LSD (Lysergic acid diethylamide), psilocybin, MDMA (3,4-methylenedioxy-methamphetamine). Higher values indicate more positive attitudes; lower values indicate more negative attitudes, *N* = 126.

SE = standard error of mean, IQR = inter-quartile range.

**Figure 1:**
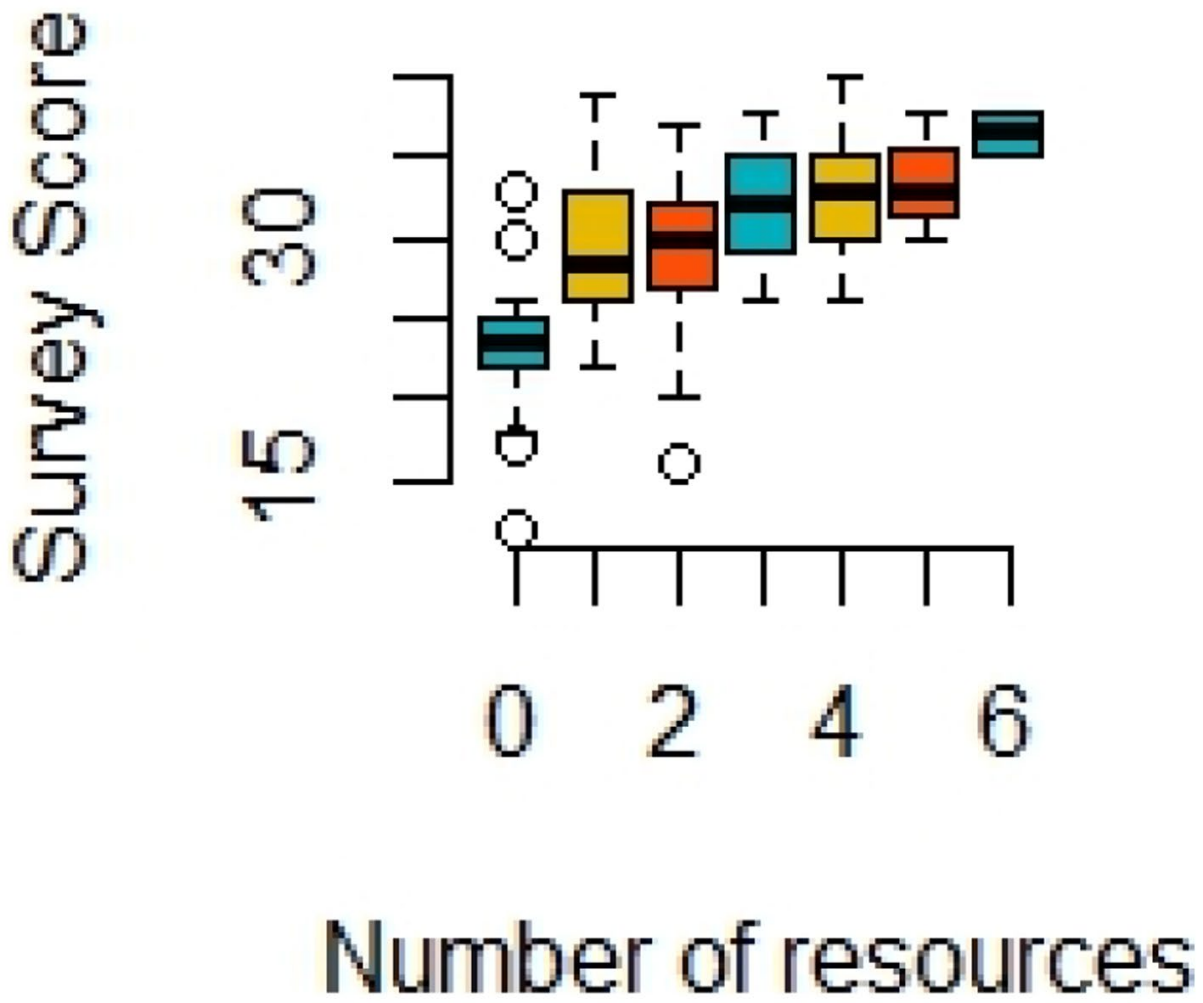
Box plot showing positive association between number of resources used to learn about medicinal uses of psychedelics and the average overall survey score (*N* = 126, Kruskal-Wallis test *p*-value < 0.0001). A simple linear regression showed that about 29% of variation in survey scores were explained by the number of resources students used (*R*^2^ = 0.288).

Results of mean survey scores for M.D. students are shown in Table 3 with higher values indicating more positive views, and lower values indicating more negative views towards the use of psychedelics in medicine. In the range of possible mean scores as 8–40 with 24 indicating overall neutral attitudes, the mean overall survey scores were 29.71 ± 5.25 (mean ± SD) with an interquartile range of 26.0–33.0, indicating overall positive perceptions toward the use of psychedelics in medicine. There were no significant differences in any of the demographic categories; however, all categorical groups generally held positive attitudes. Although not statistically significant, medical students interested in psychiatry held the highest mean score of 34.2 ± 5.23. Medical students interested in internal medicine had a mean score of 28.05 ± 4.3, which ranked the lowest of all specialties, although still overall positive. Our inferential testing showed that the only variable significantly related to overall survey scores was the number of sources used by medical students to learn about psychedelics (*p* ≥ 0.1805 in all other cases): students who used more resources showed significantly more positive views than those who used fewer sources (*p* < 0.0001, Table 3, Figure 1).

**Table 3:** Mean survey score for MD student attitudes towards psychedelics stratified by demographic factors and results of Kruskal-Wallis rank sum tests for differences in mean score between groups.

Characteristic	Mean	SD	*p*-value
**Age**			
18–24 years old	29.30	4.99	0.6265
25–34 years old	29.96	5.43	
35–44 years old	30.25	5.91	
45–54 years old	26.00	NA^c^	
**Gender**			
Male	29.92	5.03	0.5741
Female	29.48	5.48	
Non-binary/third gender	32.50	0.71	
**Race**			
White	29.95	5.44	0.3202
Black	29.50	4.95	
Asian	27.75	3.00	
Multi-racial	29.56	7.40	
Prefer not to answer	30.90	4.25	
**Ethnicity**			
Hispanic/Spanish/Latino	29.48	5.78	0.646
Non-Hispanic	29.69	5.17	
Prefer not to answer	32.33	3.51	
**Medical school**			
UNR^a^	29.42	5.02	0.1805
UNLV^b^	31.09	6.16	
**Year in program**			
First	31.18	4.68	0.2122
Second	28.74	5.34	
Third	29.04	5.32	
Fourth	30.45	5.48	
**Planned specialty**			
Emergency medicine (*n* = 17)	30.18	5.10	n/a^d^
Family medicine (*n* = 7)	30.86	6.64	
Internal medicine (*n* = 21)	28.05	4.53	
Neurology (*n* = 3)	29.67	5.51	
OBGYN (*n* = 8)	28.63	5.93	
Pediatrics (*n* = 12)	29.75	5.21	
Psychiatry (*n* = 6)	34.17	5.23	
Surgery (*n* = 20)	30.55	4.06	
Undecided (*n* = 16)	28.69	6.37	
Other (*n* = 16)	29.75	5.52	
**Number of resources used**			
Zero (*n* = 18)	23.39	4.95	*p* < 0.0001
One (*n* = 26)	29.54	4.68	
Two (*n* = 36)	29.47	4.69	
Three (*n* = 26)	31.85	3.50	
Four (*n* = 15)	32.80	4.18	
Five (*n* = 3)	33.67	4.04	
Six (*n* = 2)	36.5	2.12	

Touro University Nevada College of Osteopathic Medicine. SD = standard deviation, NA = not applicable.

a University of Nevada, Reno School of Medicine.

b Kirk Kerkorian School of Medicine at UNLV.

c Standard deviation not applicable due to sample size of one.

d Not tested due to large number of groups with small sample.

## 4. Discussion

Overall, medical students are optimistic about the therapeutic potential of psychedelics in treating psychiatric disorders, as well as displaying encouraging sentiment towards additional research efforts dedicated to investigating psychedelics. Interestingly, our study’s findings of overall positive attitudes existed despite respondents’ self-reported lack of knowledge pertaining to psychedelics, as only 40.5% agreed or strongly agreed that they were knowledgeable about psychedelics. In parallel to medical students’ intellectual curiosity for medical advancements, the lack of familiarity and knowledge regarding psychedelic-assisted therapies may demonstrate the need for educational exposure in the medical educational curriculum (26). Students who had previously utilized resources to inform themselves about psychedelics indicated stronger positive attitudes, demonstrating a directly proportional relationship between numbers of resources used and positive attitudes felt. This may reflect the changing of attitudes through increased awareness, instruction, observation, reflection, or experience (27). However, it remains important to speculate the direction of this relationship, as respondents may be using additional resources to inform their opinions or respondents may be seeking out additional information to confirm positive opinions. Thus, the role of self-selection bias in these results should be considered.

Our findings of overall positive attitudes are consistent with the positive attitudes of medical students towards psilocybin-assisted therapies (26). In contrast to medical student attitudes demonstrating low levels of concern regarding the potential adverse effects of psilocybin-assisted therapy (26), the medical students in our study were divided in their opinions regarding the possible neurocognitive risks of psychedelic-assisted therapies in general. This may be due to the broader focus of our study on psychedelics and psychedelic-assisted therapies as an overall entity, rather than a specific agent focus, thereby lumping perceived risk of all psychedelic agents into a single assessment. Given that the psilocybin-focused study had mostly osteopathic students (26), the discrepancy in concern for potential risks may also be due in part to the majority of allopathic respondents in our study: osteopathic medical curriculum incorporates the holistic approach body–mind-spirit approach to healthcare and may be inherently more receptive to the spiritual therapeutic aspect of psychedelics (26, 28).

Despite potential variation in perceived adverse effects of psychedelic-assisted therapies, the concern for adverse reactions of psychedelics is not a novel notion and has been prevalent since the 1950s (29–33). While nontoxic adverse reactions are often short-lasting (34), the incidence of prolonged psychotic reactions tend to occur in individuals with specific risk factors and overall remain rare (35). Recent studies have demonstrated that psychedelics are not associated with the development of subsequent mental disorders (36, 37) and can be safely administered in a controlled setting (38). Nevertheless, this association between psychedelics and subsequent mental illness clearly continues to affect the public perception of psychedelics, including medical students surveyed in this study. In the context of overall optimistic attitudes amongst medical students, the ambivalence towards neurocognitive risks of psychedelics may also reflect an appropriate and necessary caution regarding any new potential medical treatment under consideration.

Additionally, our study sample was a younger cohort with overall positive attitudes, and there was no effect of age on attitudes and perceptions of psychedelic-assisted therapies. This appears to starkly contrast to previously surveyed psychiatrists who believed psychedelics to be potentially hazardous and appropriately illegal for recreational purposes, although younger psychiatrists demonstrated more open mindedness towards psychedelics (12). Compared to the majority of their sample population being greater than the age of 40, our study’s sample had 95.5% under the age of 34 years or less, consistent with the typical age for individuals early in their training and consistent with the previously surveyed medical students (26).Thus, the favorable views of our younger cohort may support the generational difference noted already: the younger population may adopt more open minded views either due to increased exposure to positive information about psychedelics in media sources or decreased exposure to patients with adverse effects from psychedelics due to lack of clinical training (12, 26).

Moreover, the lack of an age effect amongst medical students towards potentially controversial treatments may not be entirely novel. A study investigating medical students’ acceptability of marijuana for treating medical conditions also found no difference according to age (39). Additionally, a similar U.K. study did not find an effect of age amongst UK psychiatrists, but did posit that a stigma towards psychedelics may be lower amongst UK psychiatrists compared to USA bound psychiatrists (21). In another study, however, cancer healthcare practitioners in both the U.S. and New Zealand agreed on the potential benefits of psychedelic-assisted therapies for cancer patients (40). It is possible, thus, that the purported difference between younger and older clinicians may reflect less of an age difference and more of a cultural variation at any moment in time. Thus, as additional clinical trial data emerges and psychedelics remain in the spotlight of media coverage, there is a strong possibility of witnessing a positive shift in attitudes towards psychedelic-assisted therapies across diverse demographic groups in various regions. Further research is required to explore the effect of age, geography, and culture on medical practitioner attitudes of psychedelic-assisted therapies.

The primary limitation of our study remains the difficulty to ascertain non-response and selection bias. Our response rate (22.7%) was lower than the 30.8% response rate of medical students captured in national email surveys (41). We cannot minimize the possibility of self-selection bias occurring amongst students who already possessed prior knowledge or positive attitudes about psychedelics; this may have contributed to an increased motivation to respond to our survey. Furthermore, there may be limited generalizability to this reported study. All participants attended a medical school in the state of Nevada with most respondents matriculating from allopathic schools. Given the differing cultures between schools and regions of the United States, results may not be generalizable to physicians-in-training in other regions of the U.S., let alone students in other countries. Nevada specifically has seen a recent bill pass in the Senate committee allowing for the legalization of psilocybin mushrooms for research purposes, suggesting a politically favorable climate in the state (42, 43). However, given that osteopathic medical in varying states of the U.S., primarily Florida, still demonstrated positive attitudes towards psilocybin-assisted therapies (26), our findings may align closely with the expected attitudes for medical students as a cohort. Additionally, medical education in Nevada currently lacks standardized curriculum content focused on psychedelics and psychedelic-assisted therapy research, and attitudes may be comparable to other institutions of similar curricula. Although overall findings may be primarily applicable to Nevadan students, this study nonetheless captures unique insight into the perspective of medical students and future professionals that may soon encounter psychedelic agents in practice. Further research is needed to explore medical students attitudes across a diverse set of institutions in a variety of regions.

Additionally, the authors recognized a pattern in which responses reflected more positive attitudes as individuals progressed from the first to the last of the eight questions in the survey. This finding may be due to answers becoming influenced by the order in which the questions were presented. By virtue of the questions asked, the survey may implicitly suggest to students that psychedelics hold research and therapeutic potential. Future research ought to randomize the sequence of questions to minimize the potential for confounders and to balance out any potential learning that forms during the survey.

Finally, in our study, the lack of significant associations between demographic variables and positive attitudes suggests that there may be no categorical difference distinguishing medical students’ attitudes towards psychedelics. Given the overall positive attitudes between medical students across all ages, sex/gender, race/ethnicity, medical school type, and level of training, there is clear optimism about the clinical potential of psychedelics. Future research efforts should explore whether the lack of categorical associations amongst medical student populations exists in additional samples. It is suggested to repeat the survey delivered and presented in this study after an interval period of several years, and amongst varied population cohorts inclusive of medical residents, to gain a greater understanding of the potential trends of the acceptance of psychedelics. Given the history and pervasiveness of stigma aimed towards both individuals with mental illness and the field of psychiatry as a whole (44, 45), an understanding of cultural attitudes towards any potentially groundbreaking treatment is imperative.

## 5. Conclusion

There is a necessity for education and communication between researchers, clinicians, patients, and the general public in reducing stigma and promoting mutual scientific understanding of the potential risks and benefits of psychedelic therapies. Although medical students are overall curious and optimistic about psychedelics, they demonstrate a lack of knowledge regarding recent research efforts and evidenced developments. As psychedelic agents may become accessible through FDA approval and through state legislative reforms in the near future, it remains essential to concentrate efforts on guidance and education for healthcare professionals. Initiating education pertaining to psychedelics at an earlier stage of training for clinicians may ease the transition between legislative reform and clinical application. It is recommended to consider incorporating psychedelic education into the standard medical education curriculum, especially as part of the comprehensive human behavior pre-clinical course and as an accompaniment section to the psychiatry clinical clerkship.

## Data availability statement

The raw data supporting the conclusions of this article will be made available by the authors, without undue reservation.

## Ethics statement

The studies involving human participants were reviewed and approved by University of Nevada, Reno School of Medicine Institutional Review Board (IRB). The patients/participants provided their written informed consent to participate in this study.

## Author contributions

IL, RF, and BT contributed to the conception and design of the study. RF created the survey instrument tool and contributed to the acquisition of data. MH performed the statistical analysis and contributed to interpretation of data. IL and RF wrote the first draft of the manuscript with IL taking the lead. BT provided oversight, wrote sections of the manuscript, and revised it critically for important intellectual content. All authors contributed to the article and approved the submitted version.

## Conflict of interest

The authors declare that the research was conducted in the absence of any commercial or financial relationships that could be construed as a potential conflict of interest.

## Publisher’s note

All claims expressed in this article are solely those of the authors and do not necessarily represent those of their affiliated organizations, or those of the publisher, the editors and the reviewers. Any product that may be evaluated in this article, or claim that may be made by its manufacturer, is not guaranteed or endorsed by the publisher.
