# Supplementary material for: Medical student attitudes and perceptions of psychedelic-assisted therapies
Source: Front Psychiatry. 2023 Jun 27;14:1190507. doi: 10.3389/fpsyt.2023.1190507 (PMC10335742; doi:10.3389/fpsyt.2023.1190507)
Supplement: Supplementary file 1 [file Data_Sheet_1.docx]

Medical Student Attitudes and Perceptions of Psychedelic-Assisted Therapies

**Appendix A**

Q1 How old are you?

- 18-24 years old
- 25-34 years old
- 35-44 years old
- 45-54 years old
- 55-64 years old
- 65+ years old
- Prefer not to answer

Q2 How do you describe yourself?

- Male
- Female
- Non-binary / third gender
- Prefer to self-describe
- Prefer not to answer

Q3 Choose one or more races that you consider yourself to be:

- White
- Black or African American
- American Indian or Alaska Native
- Asian
- Native Hawaiian or Pacific Islander
- Other
- Prefer not to answer

Q4 Are you Spanish, Hispanic, or Latino or none of these?

- Yes
- None of these
- Prefer not to answer

Q5 Which medical school do you attend?

- University of Nevada, Reno School of Medicine
- Kirk Kerkorian School of Medicine at UNLV
- Touro University Nevada College of Osteopathic Medicine

Q6 What type of medical school are you attending?

- M.D. / Allopathic
- D.O. / Osteopathic
- Prefer not to answer

Q7 What is your current level of medical school training?

- MS1 - First-year medical student
- MS2 - Second-year medical student
- MS3 - Third-year medical student
- MS4 - Fourth-year medical student
- Prefer not to answer

Q8 At this current moment, what specialty do you plan to pursue? (first choice)

- Aerospace medicine
- Allergy and immunology
- Anesthesiology
- Cardiology
- Critical care
- Cardiothoracic surgery
- Colon and rectal surgery
- Dermatology
- Diagnostic radiology
- Emergency medicine
- Endocrinology
- Family medicine
- Gastroenterology
- General surgery
- Genetics
- Geriatrics
- Hematology/oncology
- Infectious disease
- Internal medicine
- Interventional radiology
- Medical physics
- Nephrology
- Neurology
- Neurosurgery
- Nuclear medicine
- Obstetrics and gynecology
- Occupational medicine
- Ophthalmology
- Orthopedic surgery
- Otolaryngology
- Pathology
- Pediatrics
- Physical medicine and rehabilitation
- Plastic surgery
- Psychiatry
- Public health and general preventive medicine
- Pulmonology
- Radiation oncology
- Rheumatology
- Vascular surgery
- Urology
- Undecided
- Other

Q9 The following questions will ask about your attitudes towards the following psychedelic substances: 5-MeO-DMT (5-methoxy-N,N-dimethyltryptamine), Ayahuasca, DMT (N,N-Dimethyltryptamine), LSD (Lysergic acid diethylamide), Psilocybin, MDMA (3,4-methylenedioxy-methamphetamine).

The following statements are graded on a Likert scale of the following

| Strongly  disagree | Somewhat disagree | Neither agree nor disagree | Somewhat agree | Strongly agree |
| --- | --- | --- | --- | --- |

1. I would say I am knowledgeable about psychedelics.
2. The use of psychedelics increases the risk for subsequent psychiatric disorders.
3. The use of psychedelics increases the risk of long-term cognitive impairment.
4. The use of psychedelics should be illegal for recreational purposes.
5. The use of psychedelics is unsafe even under medical supervision.
6. The use of psychedelics shows promise in the treatment of psychiatric disorders.
7. The use of psychedelics may improve outcomes if used adjunctively with psychotherapy.
8. The use of psychedelics deserves further research as potential treatment for psychiatric disorders.

Q10 Which of the following sources, if any, have you used to inform yourself about psychedelics? (Select all that apply)

- News media
- Social media
- Magazine articles
- Medical Journals
- Podcasts
- Films/documentaries
- Other
- I have not used any of the following sources
